# “For a man to go to hospital, then that would be his last option”: A qualitative study exploring men’s experiences, perceptions and healthcare needs in the implementation of Universal Health Coverage in Kenya

**DOI:** 10.1371/journal.pgph.0002925

**Published:** 2024-05-07

**Authors:** Sharon N. Mokua, Lorraine Ombogo, David Mathu, Prisca Otambo, Lilian Nyandieka, Stephen N. Onteri, Schiller J. Mbuka, James Kariuki, Ismail Ahmed, Violet Wanjihia, Joseph Mutai, Zipporah Bukania

**Affiliations:** Centre for Public Health Research, Kenya Medical Research Institute (KEMRI), Nairobi, Kenya; London School of Hygiene & Tropical Medicine, UNITED KINGDOM

## Abstract

The achievement of Universal Health Coverage (UHC) requires equitable access and utilization of healthcare services across all population groups, including men. However, men often face unique barriers that impede their engagement with health systems which are influenced by a myriad of socio-cultural, economic, and systemic factors. Therefore, understanding men’s perspectives and experiences is crucial to identifying barriers and facilitators to their healthcare-seeking behaviour under UHC initiatives. This qualitative study sought to explore men’s perceptions, experiences, healthcare needs and potential strategies to inform an impartial implementation of Universal Health Coverage (UHC) in Kenya. The study employed a qualitative research design to investigate men’s healthcare experiences in 12 counties across Kenya. Thirty focus group discussions involving 296 male participants were conducted. Men were purposively selected and mobilized through the support of health facility-in-charges, public health officers, and community health extension workers. Data was coded according to emergent views and further categorized thematically into three main domains (1) Perspectives and experiences of healthcare access (2) Socio-cultural beliefs and societal expectations (3) Desires and expectations of health systems. Findings revealed complex sociocultural, economic, and health system factors that influenced men’s healthcare experiences and needs which included: masculinity norms and gender roles, financial constraints and perceived unaffordability of services, lack of male-friendly and gender-responsive healthcare services, confidentiality concerns, and limited health literacy and awareness about available UHC services. Our study has revealed a disconnect between men’s needs and the current healthcare system. The expectations concerning masculinity further exacerbate the problem and exclude men further hindering men’s ability to receive appropriate care. This data provides important considerations for the development of comprehensive and gender-transformative approaches challenging harmful masculine norms, pushing for financial risk protection mechanisms and gender-responsive healthcare delivery attuned to the unique needs and preferences of men.

## 1. Introduction

Within the context of UHC (Universal Health Coverage), the goal is to ensure that no one is left behind in that people can access quality health services wherever they are in need without being financially impoverished [1]. This inadvertently means that the health systems within which communities seek care are responsive to their needs, are well financed, well managed, and accessible [2]. Countries such as Kenya have committed to and embarked on implementing the UHC agenda, which enables nations pursuing UHC to adopt a different approach, focusing on addressing the underlying issues that create gaps in health coverage [3].

The gap in healthcare coverage majorly results from poor access to and underutilization of healthcare services in different population groups. Various studies have revealed that men are less likely to seek healthcare services than women [4,5]. Accordingly, there has been a growing recognition of men’s health issues, focusing on the trends in men’s mortality rates, especially in specific regions and demographic groups [6,7]. On average, men tend to have a shorter life expectancy than women globally, warranting increased attention to men’s specific health challenges and the disparities in their mortality rates [8,9]. So far, only seven countries globally, including only one in Sub-Saharan Africa, have developed national men’s health policies that are being used to inform on strategies to improve men’s health [10].

More recent studies highlight men’s premature deaths from non-communicable diseases NCDs and communicable diseases and morbidities related to poor health-seeking behaviours, mental health, injuries, and suicides despite men having more privileges, power, and opportunities [6,11]. In Kenya, men face the same fate, with an average life expectancy of around 61 years, compared to an average of 65 years for women [12]. The health status of men in Kenya, like in many other countries, is influenced by various factors, including socioeconomic conditions, lifestyle choices such as poor nutrition and alcohol abuse, underutilization of healthcare, and cultural norms, among others [13,14].

### Intersectionality

Biologically and socio-culturally, men’s health is shaped by a complex interplay of factors, including biological predispositions and masculine norms. Understanding the intersectionality of these influences is crucial for comprehensively addressing men’s health disparities.

Biologically, men may be predisposed to certain health risks and epidemiological findings across the globe indicate higher morbidity and mortality rates in males compared to females [15]. This discrepancy is attributed to various factors, including biological differences in inflammatory responses, sex chromosome genes, and sex hormones that shape the differential regulation of immune responses in men and women [16]. More recently, the biological differences offer insights into the mechanisms underlying the sex differences in COVID-19 mortality, emphasizing the importance of considering biological and psychosocial factors in understanding men’s higher risks of mortality [15].

Beyond biological factors, prevailing masculine ideals influence men’s adherence to certain health-related behaviours. Various studies have linked men’s behaviours and their underutilization of healthcare to gendered differences and masculine norms perpetuating the narrative of ‘Being a man’ [17–19] The societal pressure to conform to ideal masculine roles, such as being a breadwinner, and the fear of compromising traits like strength and self-reliance contribute to delays in seeking healthcare. Additionally, cultural beliefs and misconceptions about the supernatural causes of diseases influence men’s preferences for alternative medicine over formal health services in some instances [18]. These norms intersect with social stratifiers, such as age, income, education, ethnicity, occupation, relationship status, or sexual orientation, posing challenges to men’s access to health services, particularly in rural areas and among marginalized populations [18,20,21]. The lack of awareness and a shortage of tailored services for specific health needs exacerbate the problem [6,22,23].

The few studies in African countries focusing on men’s healthcare needs have revealed significant barriers and challenges hindering their access and utilization of healthcare services [6,22]. Financial constraints emerge as a significant hurdle, deterring some men from seeking healthcare when needed [6,22,24]. Furthermore, negative perceptions and attitudes towards healthcare services, often fuelled by fears of stigmatization and mistreatment, act as barriers to engagement [22,24,25]. Another pivotal theme revolves around the role of confidentiality in influencing men’s willingness to disclose their health concerns and seek appropriate care with issues around privacy preventing men’s engagement with and utilizing health services [25–27]. These findings emphasize the importance of addressing emotional readiness, stigma, confidentiality, financial aspects, and the gendered nature of healthcare settings to improve men’s access and utilization of healthcare services.

### Theoretical basis

Determining the factors influencing human behaviour is crucial to designing an effective public health program for men. Various studies have used theories, including the hegemonic masculinity and health elide theories, to understand masculinity and masculine norms and how they affect men’s health choices and behaviours [28–30]. The hegemonic masculinity (HM) theory outlines the idealized form of masculinity within a culture, dictating prescribed traits and behaviours that men strive to achieve [31]. The HM theory offers insights into how cultural ideals of masculinity shape men’s health behaviours, providing crucial context for understanding why men may prioritize conformity to societal norms over their health [32,33]. This framework acknowledges the multifaceted nature of masculinity, considering intersecting factors such as age, socioeconomic status, and cultural norms, thereby enabling a nuanced analysis of the determinants of men’s health choices. Complementarily, health belief theory provides a structured approach to identifying barriers and facilitators influencing men’s engagement with healthcare services, including perceived benefits, barriers, and self-efficacy [30,34]. By integrating these theoretical perspectives, this paper deepens the understanding of men’s health behaviours.

### Our research

Most literature in Africa has focused on men being gatekeepers to women’s health, focusing more on men’s involvement as fathers and partners in healthcare [35–38]. Research efforts in low- and middle-income countries have largely overlooked men as independent healthcare seekers, resulting in a paucity of studies that explicitly examine men’s autonomous healthcare access needs and experiences [18]. Financing strategies and considerations have dominated the current discussion on UHC, but community engagement is equally essential for priority setting and determining which interventions can be effectively delivered to specific populations, thus bringing us closer to achieving health equity [39,40]. Community engagement offers an opportunity to address shortcomings in the healthcare policy, allowing us to rethink the approach to determinants of health, gender equity, and, in the process, how men are positioned at the receiving end of healthcare services [41]. Involving men as active users of healthcare services is crucial for the movement towards equitable and inclusive health for all as per the aspirations of Universal Health Coverage [24,42].

To implement a truly universal UHC scheme, we cannot leave out conversations around gender and norms, including the type of coverage offered, how health within a specific context is defined, who is included, and if equity is ensured in every step [43]. It is pertinent to make continuous efforts to understand the real-time and persistent, if any, healthcare access challenges driven by both demand and supply side factors that reflect today’s health systems as we work towards better health systems of tomorrow for men. Accordingly, this study sought to explore the male population’s perceptions of healthcare, bringing along experiences and interactions men have had with the current health system in Kenya and documenting the efforts and strategies that can be employed towards realizing equity in UHC for all in Kenya.

## 2. Methods

### 2.1 Study design

The qualitative study was conducted as part of a larger mixed methods study investigating population needs and health system capacity in the context of the implementation of UHC in Kenya. This particular paper draws upon data from the qualitative arm which used focus group discussions (FGDs) to explore and understand men’s lived experiences with accessing healthcare in their communities. Thematic analysis was then employed to identify, analyse and draw out valuable themes and findings from the data.

### 2.2 Study setting

The study was conducted in 12 out of 47 Counties in Kenya. They included Bomet, Bungoma, Homabay, Isiolo, Kisumu, Kitui, Machakos, Meru, Nyandarua, Nyeri, Taita Taveta and West Pokot.

### 2.3 Study participant sampling and recruitment

Male participants for the FGDs were purposively selected and mobilized through the support of health facility-in-charges, public health officers and community health extension workers (CHEWs) using the pre-set inclusion criteria (Men aged between 20 and 60 years, willing to share their experiences and residing in the Community Health Unit registered households that are situated around the different levels of targeted health facilities). This ensured that we included probable healthcare service users. The recruitment of study participants was conducted between February 2020-March 2020 and October 2020-December 2020.

### 2.4 Sampling of study sites

All Twelve counties were purposively selected to capture diverse experiences with the inclusion of 4 pilot counties (Kisumu, Nyeri, Machakos and Isiolo) where the UHC program piloted and 8 non-pilot counties (Bomet, Bungoma, Homabay, Kitui, Meru, Nyandarua, Taita Taveta and West Pokot). The selection of pilot counties was based on an already pre-determined criterion by the Government of Kenya due to the listed factors: a high burden of; i) Communicable diseases; ii) Non- Communicable Diseases (NCDs); iii) Vulnerable and migrating populations (Pastoralists) iv) Road traffic injuries. The pilot counties were each clustered with two non-pilot counties that included one neighbouring county and one distant county creating a total of 4 study clusters. The neighbouring counties were those with similar socio-economic and population dynamics and the possibility of being affected by the UHC rollout in the pilot count. Distant counties with similar socio-economic and population dynamics were categorized as those being far off from pilot county where they were unlikely to have UHC intervention influence.

### 2.5 Data collection, management and analysis

A total of 30 FGDs among men were conducted using a structured study guide (see S1 File Structured FGD guide for Men). The FGDs each consisted of between 8–10 participants with an overall participation of 296 men. The FGDs were conducted in appropriate, convenient and conducive venues with discussions moderated by social research scientists with qualitative data collection expertise backed up by note-takers. The FGDs were conducted using the national Kenyan language (Kiswahili) or English and in a few situations where the use of local language was necessary, local certified translators were engaged to support the translations during the discussions. The discussions lasted between 60 and 90 minutes with reimbursement of transport costs amounting to Kenya Shillings 500 (Approximately 5 USD) per participant. All discussions were audio-recorded and backed up with note-taking. Data was transcribed verbatim and translated into English where necessary. The data was anonymized using codes for the different male population groups according to the study site. Researchers engaged in self-reflection throughout the research process. Familiarization of the data was done by members of the research team and issues arising from transcription and translation were addressed in real time. The team developed an initial thematic framework for the deductive manual coding guided by the study tools and objectives and inductively where subsequent themes were developed to better understand and articulate men’s perceptions as well as overall experiences with healthcare and their health-seeking. This was followed by a second round of refinement of the themes through quality cross-checks and consensus [44]. In some cases, data was re-categorized and/or merged. During thematic development, extensive self-reflection was performed to ensure that the themes developed were grounded in the data.

In this study, we sought to enhance transferability by providing a detailed description of the research setting, the characteristics of the study participants (men aged 20–60), and the context in which data was collected. We also employed purposive sampling to ensure diversity among participants. To enhance dependability, we documented every step of the research process This included maintaining focus group transcripts and documenting methodological decisions. By doing so, we created a clear and transparent record of the research process, allowing for potential replication and assessment of the dependability of our findings. We also used triangulation by collecting data through multiple methods (individual interviews and focus groups within the larger study) and involving multiple researchers in the analysis to reduce the potential for bias and errors to enhance the credibility of our interpretations. We also ensured that data analysis was systematic and that interpretations were grounded in the data by using direct quotes from participants to support our assertions. The study findings are presented in narrative form and supported by verbatim quotes. This study is reported in line with the Standards for Reporting Qualitative Research (SRQR) Checklist (see S2 File)

### 2.6 Ethical considerations

Before conducting this study, Scientific and Ethical approval to conduct this study was obtained from the Kenya Medical Research Institute’s Scientific and Ethical Review Unit (SERU)(KEMRI/SERU/CPHR/005/3945). The Research permit was obtained from the National Council of Science, Technology & Innovation (NASCOTI), while written authorization to conduct the research in the various counties was obtained from County Health Directors. This study also sought a letter of support from the State Department of Health, Ministry of Health (MOH). Informed consent to participate in the study was obtained in written form (signing or thumbprint) from all participants in a language well understood to them prior to their participation in the study. To ensure confidentiality and anonymity, we did not collect any personal identifiers such as names and discouraged discussion of personal views outside the FGD setting.

## 3. Results

### 3.1 Demographic characteristics of participants

Table 1 shows the number of participants interviewed by their different socio-demographic and socio-economic characteristics. The men interviewed were between the ages of 20 and 60. Most of the respondents were farmers with a majority having obtained education up to Secondary level.

**Table 1:** Sociodemographic characteristics of the male FGD participants.

Characteristics	Total Number of Respondent	Percentage
**Age (years)**		
20–29	32	10.8%
30–39	93	31.4%
40–49	92	31.1%
50–59	59	19.9%
60	20	6.8%
**County of Residence**		
Bomet	37	12.5%
Bungoma	20	6.8%
Homabay	38	12.8%
Isiolo	29	9.8%
Kisumu	21	7.1%
Kitui	10	3.4%
Machakos	10	3.4%
Meru	40	13.5%
Nyandarua	39	13.2%
Nyeri	32	10.8%
Taita Taveta	10	3.4%
West Pokot	10	3.4%
**Education Level**		
No Education	9	3.0%
Primary level	101	34.1%
Secondary	141	47.6%
Post-Secondary	45	15.2%
**Occupation**		
Farmer	182	61.5%
Business	58	19.6%
Casual Work	3	1.0%
Employed/Professional	27	9.1%
Student	3	1.0%
Self- employed	8	2.7%
Spiritual leader	5	1.7%
Unemployed	10	3.4%

### 3.2 Qualitative findings

The findings focus on “What it means to be a man seeking care in the local health system” and “What men want from the healthcare systems” The study findings are categorized into 3 main themes with outlined subthemes as summarized in Table 2. This is followed by a detailed description of the findings in subsequent sections with the inclusion of selected quotes from the qualitative transcripts to support the key findings.

**Table 2:** Summary of Themes and Subthemes identified from the thematic analysis.

Themes	Subthemes
1. Perspectives and experiences of healthcare access (how men perceive, access, and interact with healthcare services)	Affordability of health service-related costs
	Health service operations- *unfavourable facility operating hours and long waiting times*
	Physical infrastructure at facilities-*limited private male friendly spaces*
	Interactions with providers-*unprofessionalism of healthcare providers*
	A gap in men’s knowledge and familiarity with healthcare
	Gender disparities in program focus
2. Socio-cultural beliefs and societal expectations (common perspectives that men may have regarding cultural norms related to health seeking)	Traditional masculine norms
	Socio-cultural beliefs and practices *limited male healthcare providers (gender concordance*)
3. Desires and expectations of healthcare systems (common needs and preferences that men expressed regarding their interactions with health systems)	Male-specific mental health support and programs
	Male-specific confidential Sexual reproductive health (SRH) services
	Male-specific screening services
	Outreach programs that meet men where they are.
	Desire for Gender-concordant healthcare i.e. access to male providers
	Male specific clinic days

### Theme 1: Perspectives and experiences of healthcare access

#### Affordability of health service-related costs

The affordability of healthcare emerged as a prominent concern, as men with limited income felt burdened by the high costs associated with accessing essential healthcare services. The burden of out-of-pocket payments compels men to forgo health services, as they are forced to prioritize multiple familial responsibilities over their personal healthcare needs as shown in the quotes below.

*“A man may be unwell together with the wife and family and because he doesn’t have money he will pay for their bill and stay unwell because of shortage of money*.” (*FGD 016 Kitui)*

***“****They don’t have money to go to hospital to fully understand the problem and get a solution*. *In a month he can only get 500 shillings and he has so many needs like school fees for their children therefore he can’t go to hospital since he has other responsibilities*. *Also*, *many of them are scared of people knowing their situation*.**”** *(FGD 008 Bungoma)*

Worth noting is the challenge of paying premiums for national health insurance schemes like National Health Insurance Fund (NHIF) particularly for men with limited resources.

“NHIF here in the rural area is a challenge because it costs 500 per month but there are people who even don’t get 200 shillings per month. So it becomes a challenge for a man to enrol his family to NHIF since they use the money to buy a tin of maize instead. Therefore, those with NHIF are few compared to those with NHIF.” (FGD 008 Bungoma)

In addition, the cost of accessing health services was more pronounced among men in lower socioeconomic groups who are mostly engaged in skilled and unskilled low-wage labour.

***“****Another problem that men face in this area is most of them work as jua kali* (low wage skilled or unskilled labourer*) so they get body pains and when one gets sick*, *he won’t bother to go to hospital because he knows it may be too costly so he passes by the shop and buys painkillers and treats himself*.” *(FGD 007 Bungoma)*

Due to the high costs associated with healthcare services, some men reported resorting to self-treatment or traditional medicine, which was perceived as more viable and affordable compared to contemporary treatment options, especially when faced with competing family responsibilities or a combination of different financial challenges but ultimately, they said “for a man to go to the hospital, it was usually his last option”

*“Men have challenges*. *They give health priority to their wives and children*. *It is very rare for men to seek health services at a health facility*. *They will instead turn to herbal treatment but for a man to go to the hospital then that would be his last option*” *(FGD 013 Isiolo)*

***“****Many will come since a lot of us fall sick at home but only seek traditional medicine because there are no other alternatives*. *We don’t have money to go to hospitals and we are forced to seek traditional alternatives so as just to feel better … Because they think the traditional one is cheaper*. *A glass is 50 shillings and the same amount is for a registration book here and still they’ll need more for lab services and such*. *So they opt for traditional medicine instead*” *(FGD 008 Bungoma)*

#### Health facility operations

Participants expressed concerns about the operating hours of health facilities, which often conflict with their income-generating activities during the same hours, opting to forgo treatment to continue working on weekdays. Additionally, men reported experiencing long waiting times due to a large patient population and a limited number of healthcare providers.

*“The one main challenge is this*, *and will not be afraid to speak is that we would like to ask the government to help us by making this facility to be operational even on weekends*. *You know weekends doesn’t spell the end for sickness*. *Sickness pops up anytime*. *So even weekends we ask for quality healthcare*. *I know we all have to rest but if there are more than one doctor at the hospital*, *let’s say 2 or 3 then they can be changing shifts*. *There is also a challenge of service at night*. *Catching malaria at night is hectic since service is very poor* (*FGD 006 Bungoma)*

*“The population here is usually very big but there is only one doctor*. *The patient ends up on the queue for almost 2hours which is an offence*. *Remember the patients still have to pay something what if it becomes free of charge and still there is only one doctor*. *It will be very challenging and will require one to seek an alternative*. *This will end up not being a free service*.” *(FGD 006 Bungoma*)

#### Physical facility infrastructure

The provision of services for different groups at single or shared service points, rather than having separate spaces, evoked feelings of discomfort among men due to the perceived lack of privacy during patient-provider consultations on sensitive issues such as sexual and reproductive health (SRH). Additionally, men cited instances where waiting rooms were in close proximity to consultation rooms, compromising confidentiality, as well as situations where they had to sit side by side with women while queuing for services.

*“We wanted to say that privacy we look at it in two ways*. *There is that privacy of your conversation with the doctor which is good*. *The other privacy is in terms of the waiting room which is so close to the consultation room and squeezed such that a patient waiting on the bench can hear everything*. *When you leave the hospital*, *you will hear the news everywhere not because the doctor said so but because the next patient waiting could hear everything*. *The confidentiality of doctors on patients’ information is good but privacy during consultation is a challenge since the facility is squeezed*. *Privacy for men seeking confidential health care especially for STDs and STIs*.” *(FGD 005 Bungoma)*

*“Someone should go to the hospital but it’s always hard when you go there and find women on the queue and also*, *they feel embarrassed to go and be told that they have this disease*, *which brings stress to men*.” ***(****FGD 026 Nyeri)*

#### Interaction with healthcare service providers

*The professionalism of healthcare workers*. There were concerns about the lack of professionalism, particularly during interactions with healthcare providers. Specific issues mentioned included poor reception, harsh treatment, and the unauthorized sharing of patient information by healthcare providers.

*“The staff attitude and unwillingness to serve people*… .*If we get nurses who do not listen to patients then all of them will stop going to that facility and seek services elsewhere*…*Poor reception of patients in the facility*.(*FGD 015 Kitui)*

*“Some (Healthcare providers) do not have privacy*. *They spread information about patients*.” *(FGD 012 Homabay)*

#### A gap in men’s knowledge and familiarity with healthcare

The lack of awareness about healthcare services, including universal health coverage (UHC), contributes to men’s limited understanding of the benefits of seeking medical care. Some men also expressed the need for general awareness programs and educational initiatives to increase knowledge about common diseases, symptoms, and the importance of early intervention.

*“What I can add is that if possible UHC should do mass education for people…Because to be honest we men are very ignorant about our health…Yes and truth be told*, *if we even look at the time we got here*, *I don’t think there is a man who has come to the hospital*. (*FGD 028 Nyeri)*

*“If I can add that something that is very much needed in our area*, *it is education*, *education has to sensitize and motivate people to change*, *but they still have the culture or custom*, *especially with issues to do with hospital*.” (*FGD 030*, *West Pokot)*

#### Gender disparities in program focus

Men felt that the current healthcare systems have been designed around women and children with minimal program focus created around their own unique needs. This elicited feelings of “Being left out” even where UHC had been piloted to offer free services to all. They highlighted that they majorly see plenty of female services at facilities and fewer male services.

*“Mostly only women’s services are available like clinics but men’s services maybe you just come*, *see a doctor but you don’t get medications so many people especially men no longer come to seek services here*. *We don’t get the right services that we would want so we go to other places*…*Maybe you go to a traditional doctor*…*/Yes*. *Roots because there are no medications at the facility that is why we chew roots*” (*FGD 013 Isiolo)*

*“We want the government to register men with Linda Wazee (Care for men) just like women are registered with Linda Mama (Care for women) …Just like women get services using the NHIF card we also want the government to help men get services*. *We are one family so men should be given free services just like women*.” (*FGD 018 Meru)*

*“I was thinking that the government should use the UHC to effectively sensitize locals about cancer and also focus on cancer that generally affects men*. *If you look at our notice boards*, *the government has been focusing more on women yet cancer is a serious disease which affects both genders*. *We don’t have any machines for screening cancer and we discover such diseases very late yet that is not the case with women*.” (*FGD 005 Bungoma)*

### Theme 2: Cultural norms and societal expectations

#### Traditional masculine norms

The notion of masculinity and the tendency to be stoic or tough can influence men’s healthcare-seeking behaviour forcing them to minimize their health concerns. Some men mentioned persevering through illnesses, such as malaria, without seeking medical attention because they perceive certain ailments as minor or believe that men should endure and manage their health issues independently.

***“****Men do a lot of work but when they can still walk*, *they think they don’t need to seek medical attention and when they need to go to the hospital*, *they may not have the means to come to the hospital*. (*FGD 029 Taitataveta)*

“*When we get malaria as men*, *we persevere in the house without going to hospital because we think malaria is such a small thing for us to come and pay to be treated*. *Most of us when we are sick*, *we think it’s better we die as men*.” (*FGD 009 Bungoma)*

#### Socio-cultural beliefs and practices

Traditional gender roles that are perpetuated by culture influence men’s interaction with healthcare. Some men spoke on the adherence to the belief that it is socially unacceptable for them to undress or discuss intimate issues with female healthcare providers, especially if they are younger. These traditional norms create barriers and discourage men from seeking healthcare or disclosing their health problems in an expressive and non-judgmental environment.

*“Now if you come here and say that*, *you feel challenged because the person you are telling looks like your child…*. *So you feel embarrassed to remove your…or them to have a proper look so that is one problem*…*What I am saying*, *traditionally it is not good for any woman*, *whoever it is*, *for a man to undress and show her his private parts*. *So on that*, *there is a big problem*. *There is a big problem for the old men to give all their problems*. *If you find a woman*, *he can pretend that he is not sick and goes back*” (*FGD 019 Meru)*

*“I came to the hospital when I had leg pain*. *I was served well but there was a person who had an STI (*Sexually Transmitted infection) *and it was hard for him to explain to female healthcare workers his problem*. *He decided to lie that he had a headache so that he can be treated*. *The hospital should be gender equal so that they can help such people*. *I was forced to intervene but it was late and he had left*” (*FGD 001 Bomet)*

Men expressed a fear of vulnerability that sometimes extends to sensitive issues like erectile dysfunction, and prostrate issues among others. Men may be hesitant to seek help for conditions perceived as socially sensitive, fearing judgment as this may be tied to stigmatization and shame. They mentioned that they would rather avoid talking about these issues but sometimes preferred talking to a male doctor who could potentially help.

*“What I can say is that we should focus on STDs(*Sexually Transmitted Diseases). *Some very many men suffer from these diseases but are forced to stay at home*. *They are embarrassed about these diseases*. (*FGD 007 Bungoma)*

### Theme 3: Desires and expectations of the healthcare system

#### Need for male-focused community health services such as

*Mental health support and programs*. Men recognise the importance of addressing mental health issues and believe that counselling and the availability of community mental health support services could help individuals who are struggling. They emphasized on the need for the creation of inclusive spaces where they could openly discuss their mental health and emotions, challenging the historical expectation for men to deal with their emotions on their own.

*“We need counselling and outreaches in the community*. *Recently*, *we heard there was someone who committed suicide*. *Would he have been counselled*, *he would have been helped*.” *(FGD 003 Bomet)*

*“Another thing is that men do not disclose those issues if they have them so I would say that they bring them counselling services so that they can be counselled*” *(FGD 021 Meru****)***

The men also recognized the connection between physical and mental health. They felt that they often face challenges and pressures at home that may impact their mental health, leading to physical health issues. They proposed provision of dedicated counselling specialists to help them address these challenges, provide support, and promote overall mental well-being.

*“These problems that men have*, *when people grow old*, *they have difficulty urinating*. *I would also like universal health care to address that because there are some people who when they get to sixty years and above*, *that problem really affects them -I went to one doctor and he told me*. *It is like depression*. *It is what causes men to sometimes have difficulty with that thing*… *So what I would ask is that we have a counselling specialist…Yes*. *Because men are depressed at home*, *but you wouldn’t know if they are depressed and that problem gets to the body*. *So*, *when they go for that job*, *he gets defeated…Yeah*. *Because of Mental health problems*.” (*FGD 020 Meru)*

*Confidential Sexual reproductive health services*. Men highlighted a need for confidential male SRH services. They mentioned the importance of private and confidential physical spaces during consultations, additionally advocating for family planning options for men, such as vasectomy, they also emphasized the need for private SRH services for both men and their partners.

*“I think when the program will be rolled out*, *there will be enough rooms for privacy for men seeking confidential health care especially for STDs and STIs*. *In such rooms I will be able to talk freely to the doctor about my needs…I can say if I have an STD as a man and I have come to be treated*, *then there should be free treatment for men and their partners in cases of STDs*.” *(FGD 005 Bungoma)*

*“I think there should be more buildings because we don’t even have counselling room*, *the rooms are 2*, *so when a man comes with his partner there is no secrecy because everyone is listening to the discussion and some diseases are private*” (*FGD 029 Taita-Taveta)*

*Health screening services*. Similar to health screening services for women such as cervical cancer screening, men expressed the need for screening services for conditions that affect them too. They recognized the impact of these conditions on men’s health and well-being, and believe that early detection and prevention through screening can help address these issues effectively. Men also expressed the importance of timely and efficient healthcare services, with shorter waiting times, to ensure that they receive quality care in a timely manner.

***“****Because as we speak*, *this is a level two facility*. *If you go to this dispensary*, *women are screened for cervical cancer yet me if am in pain down there*, *I have to go to Kenyatta or a level four facility*. *So that is a good health need for men*. *Campaigns should be done for non-communicable diseases and our facilities should be equipped with such things*. *That’s a point*.” (*FGD 005 Bungoma)*

*“I think the first service that should be prioritized is for chronic illnesses*…*Like diabetes*, *hypertension*… *They are really affecting men and even lead to this potency issues since they always start from somewhere*. *I ask on behalf of men that these chronic illnesses be given first priority…We as men are really suffering from cancer so if the government does bring this program*, *then they must focus on it*… *If men are to get quality services here then we must focus on time*. *He should be attended to in between 30 to 40 minutes and released to go home*” (*FGD 011 Homabay)*

*Outreach programs that meet men where they are*. To feel equally supported and included in programs, men shared the need to also be served with outreach community services that are created and targeted specifically for them.

“*If healthcare providers can go to men where they stay and talk to them and know what problems they have it will be good*. *Why do I say this is because the women are really being taken care of more than men in the community*” (*FGD 014 Kisumu)*

#### Desire for gender-concordant healthcare

Men expressed a desire for gender-concordant healthcare providers, particularly in the context of reproductive health services. Many participants acknowledged a preference for male healthcare providers when seeking care similar to what is available for women.

*“There is need for a male nurse here for us men just like in police station where there is gender basic for women*.” (*FGD 016 Kitui)*

*“There should be equal male and female healthcare workers*. *When you come and find only women*, *there are some questions you can’t ask*. *If you find a man*, *it is easier for you to explain*.” (*FGD 003 Bomet)*

#### Need for male-specific clinic days

Men, just like women, face health challenges some of which may require tailored support and intervention. In the context of this study, men expressed a need to have male-specific clinic days same way women have pre-and post-natal clinic days as supported by the quote below:

*“Could it be possible that we get clinic days for the men so that we can meet the doctors and explain our problems to them*? *We also ask a room in our hospital where we men can have a conversation with fellow men doctors and get treated from since some diseases are only for men and it is very difficult for men to explain them to female doctors*”. (*FGD 017 Machakos)*

## 4. Discussion

This study found that one of the major reasons for men not accessing healthcare services was as a result of health-related costs coupled with financial constraints linked to out-of-pocket payments for services, premiums for insurance payments and competing household responsibilities. In reviews done by Roudsari and Macdonald, one essential component of conventional masculine identity is the position of provider. In this specific role, men majorly take on household financial responsibilities which sometimes causes them to forego their health-related costs because of their socio-economic disadvantage. Men with less financial resources avoid interacting with health services tied to a cost implication to put their family members’ needs first [45,46].

Another study done in Kenya noted that many of the respondents, which included men, did not have health insurance and/or were not able to make payments on premiums to cover health insurance which necessitated out-of-pocket expenditure on health services [47].

Therefore, addressing financial barriers, such as implementing free services, insurance coverage or subsidies, could potentially improve access to healthcare for men with limited resources [24].

Standard health facility operating times and long waiting times were some of the challenges reported as presenting health access barriers for men. Other similar studies have discussed that seeking services are perceived as more burdensome for self-employed men who depend on day-to day work for their wages. Alike, it is beneficial for a man to work during normal working hours as opposed to seeking health services during these same hours as this presents an economic trade-off. Whether it is taking time off for a full day or spending extended time waiting for healthcare services, there is the risk of lost wages [48–52].

The lack of designated private and confidential spaces for service delivery, as opposed to general shared areas catering to all population groups, emerged as a significant deterrent for men seeking healthcare in this study. This finding is consistent with several other studies that have highlighted privacy and confidentiality concerns as barriers to men’s utilization of health services, particularly for sensitive health issues. [24,27,53]

The absence of separate, male-oriented spaces within healthcare facilities can create an unwelcoming and uncomfortable environment for men. Having to access services in open, general areas alongside female patients can heighten men’s sense of vulnerability, inhibiting their willingness to disclose personal health information or discuss delicate matters openly. This is especially pronounced when seeking care for issues related to sexual and reproductive health, mental health, or other conditions perceived as sensitive. In a program implemented in Tanzania, in addition to training providers to offer male-friendly health services, the program established and promoted male corners to serve as safe spaces for men that offer an integrated, male-friendly service package responding to multiple health needs at the same time, showing that private and confidential healthcare spaces can enhance men’s comfort and willingness to seek care [54]. Ensuring private and confidential healthcare services can enhance men’s comfort and willingness to seek care. Healthcare facilities can consider redesigning spaces and workflows to address these privacy concerns and create a more supportive male-friendly environment [27,55]

Another notable finding was the lack of professionalism by the providers with instances of poor reception, harshness, and breaches of patient confidentiality. This has been echoed in other studies where health provider attitudes have been found to negatively significantly impact men’s experiences of access to healthcare. Our findings align with those of previous studies in Sub-Saharan Africa [56]. Positive interactions between male patients and healthcare providers have been shown to create spaces where men seek and receive care willingly and interactively [57,58]. Professional training programs focusing on patient-centred care and empathy can help improve provider-patient interactions and enhance the overall healthcare experience for men.

Moreover, men reported uncomfortable encounters while seeking SRH treatment services from female healthcare professionals. The desire for more male healthcare providers, especially for sexual and reproductive health services, indicates a need for a gender-sensitive approach. The study findings further suggest that having access to male healthcare professionals who are knowledgeable and non-judgmental can contribute to a more comfortable and open healthcare environment for men [59]. Contrary to this opinion expressed by a majority of men in the discussions, some of them did not perceive the gender and age of the provider as a hindrance to receiving care, indicating a diversity of perspectives on this particular issue.

The findings from this study highlight gender disparities within health programs and systems that have traditionally prioritized women’s and children’s health. The disproportionate focus on maternal, newborn, and child health programs has undoubtedly contributed to significant improvements in these areas. However, it has also led to a relative neglect of men’s healthcare needs, perpetuating gender disparities and hampering progress towards achieving universal health coverage. Participants expressed a pervasive sentiment of "being left out," stemming from the perception that healthcare services and facilities were primarily designed and tailored for women and children. This aligns with previous research that has identified institutional biases and gender-blindness in health policies and service delivery models where limitations observed have centred around organisational policy, practice and gendered expectations [27,60]. Men’s perceptions of being overlooked by healthcare systems can discourage their engagement with services, exacerbating existing barriers to healthcare access and utilization.

Several papers argue that men’s health-seeking behaviours are often related to how society constructs gender identities and roles for men and women—whether concerning family responsibilities, work-life, or accessing health services. In other words, men’s risk-taking behaviours and underutilization of health services strongly stem from gender differences and predominant masculinity norms defining what it means to be a "man" perpetuating stoicism and self-reliance [6,61,62].

Men further expressed discomfort and challenges in seeking help for issues related to sexual and reproductive health due to the presence of female health seekers and interactions with female healthcare providers. This finding aligns with previous evidence that highlights the impact of gender dynamics and cultural norms on men’s help-seeking behaviour [63].

Another important finding underscores the critical role of health literacy and awareness in shaping men’s healthcare access and utilization of appropriate interventions. A study by Smith et al. [64] highlighted the lack of adequate knowledge on health issues as a significant barrier preventing men from seeking and accessing suitable healthcare services. This gap in health knowledge and awareness among the male population aligns with the evidence presented by Hardee et al [65], revealing that men and young adolescents often receive inadequate information and services through existing health programs. Consequently, they may lack the necessary knowledge and skills to navigate and make informed decisions about their sexual and reproductive health needs effectively. Finally, the present study’s findings align with existing literature by drawing attention to critical areas of healthcare that have historically lacked sufficient emphasis and resources when it comes to meeting the needs of men. Specifically, participants highlighted gaps in mental health support, sexual and reproductive health services, and screening for non-communicable diseases (NCDs) as domains that require focused attention in anticipation of Universal Health Coverage (UHC) implementation [66]. The government must therefore ensure they understand the most effective and efficient ways to reach men ensuring that UHC strategies and information meaningfully engage men ‘where they are’

## 5. Conclusion

This qualitative research unveiled invaluable insights into the multifaceted sociocultural, institutional, and individual factors influencing men’s healthcare experiences, perceptions, and needs, underscoring the challenges and opportunities inherent in achieving Universal Health Coverage (UHC) objectives. The findings accentuate the imperative for continued exploration of gender dynamics in healthcare access and utilization, while emphasizing the significance of adopting gender-sensitive and inclusive UHC implementation approaches. The study’s far-reaching implications highlight the need for gender-transformative interventions, gender-responsive healthcare delivery models, and concerted efforts to challenge societal norms perpetuating healthcare as a ’feminine’ domain. Additionally, addressing financial barriers, promoting open discussions on men’s health, and establishing continuous evaluation and feedback systems for men and boys is crucial. As healthcare systems evolve towards realizing UHC and "Health for All" ambitions, incorporating these insights into policies, practices, and interventions will create equitable and inclusive healthcare environments that acknowledge and address the distinct needs of diverse populations, including men, ultimately promoting overall societal well-being.

## 6. Implications for policy

The findings of this study can help influence the development, modification and implementation of policies, regulations and guidelines for men and boys. Considerations may include developing and implementing gender-transformative healthcare policies such as those that support educational programs to raise awareness about men’s health issues in schools and communities. Policymakers should consider strategies to increase the representation of male healthcare providers, particularly in specialities related to reproductive health. This may involve targeted recruitment efforts, educational initiatives, and specialized training programs to ensure an adequate supply of male healthcare professionals in these areas. Healthcare facilities should develop guidelines and protocols that accommodate patients’ preferences for gender-concordant care, where feasible and implement specialized male-focused healthcare programs and initiatives focused on addressing men’s physical and mental health needs. These programs could include screening and treatment services for conditions prevalent among men, such as cardiovascular diseases, prostate cancer, and mental health issues like depression and substance abuse. Also, investing in research and data collection efforts to inform evidence-based policies, and explicitly integrating men’s health into the overall UHC framework and strategies will be crucial. Lastly, there is a need to allocate resources to fit, expand and enhance the health system’s capacity to address men’s health issues and ultimately support men in managing challenges and living a long healthy life.

## 7. Study strengths and limitations

This study was conducted only in 12 out of the 47 counties in Kenya, and among a few purposively sampled male community members. Their views may therefore not necessarily represent the opinions of all men and UHC. While this limitation is an expected nature of qualitative studies, the findings cannot be generalized to other populations and settings, since community needs and health system gaps are to a large extent context-specific. However, the lessons and opportunities that the findings from this study present can be applied to other settings in Kenya and Sub-Saharan Africa where there exist similar health system characteristics.

## Supporting information

S1 File Structured FGD guide for Men. (DOCX)

S2 File Standards for Reporting Qualitative Research (SRQR) checklist. (DOCX)

## References

[1] Universal Health Coverage (UHC) [Internet]. World Health Organization; 2023 [cited 2023 Jul 15]. Available from: https://www.who.int/news-room/fact-sheets/detail/universal-health-coverage-(uhc)

[2] Levesque JF, Harris MF, Russell G. Patient-centred access to health care: conceptualising access at the interface of health systems and populations. International journal for equity in health. 2013 Mar 11;12:18. doi: 10.1186/1475-9276-12-18 ;PMCID: PMC3610159. 23496984 PMC3610159

[3] Reich MR, Harris J, Ikegami N, Maeda A, Cashin C, Araujo EC, et al. Moving towards universal health coverage: lessons from 11 country studies. The Lancet. 2016 Feb 20;387(10020):811–6. doi: 10.1016/S0140-6736(15)60002-2 26299185

[4] Affleck W, Carmichael V, Whitley R. Men’s mental health: Social determinants and implications for services. The Canadian Journal of Psychiatry. 2018 Sep;63(9):581–9. doi: 10.1177/0706743718762388 29673270 PMC6109884

[5] Rice SM, Oliffe JL, Kealy D, Seidler ZE, Ogrodniczuk JS. Men’s help-seeking for depression: Attitudinal and structural barriers in symptomatic men. Journal of Primary Care & Community Health. 2020 May;11:2150132720921686. doi: 10.1177/2150132720921686 32410489 PMC7232115

[6] Etienne CF. Addressing masculinity and men’s health to advance universal health and gender equality Rev Panam Salud Publica. 2018;42:e196. Available from: doi: 10.26633/RPSP.2018.196 31093223 PMC6386147

[7] Robertson S, Baker P. Men and health promotion in the United Kingdom: 20 years further forward?. Health Education Journal. 2017 Feb;76(1):102–13.

[8] Griffith D.M., Bruce M.A., & Thorpe Jr. R.J. (Eds.). (2019). Men’s Health Equity: A Handbook (1st ed.). Routledge.

[9] Jack L Jr, Griffith DM. The health of African American men: implications for research and practice. American journal of men’s health. 2013 Jul;7(4_suppl):5S–7S. doi: 10.1177/1557988313490190 23708877 PMC5178829

[10] Baker P. Delivering Men’s Health: A Guide for Policymakers and Service Providers. Global Action on Men’s Health; London (UK). 2021

[11] Men’s Health [Internet]. World Health Organization; 2018 [cited 2023 Jul 15]. Available from: https://www.who.int/europe/news-room/fact-sheets/item/men-s-health

[12] Life expectancy at birth, total (years)—Kenya [Internet]. World Bank; [cited 2023 Jul 15]. Available from: https://data.worldbank.org/indicator/SP.DYN.LE00.IN?end=2021&locations=KE&name_desc=true&start=1960&view=chart

[13] Kendagor A, Gathecha G, Ntakuka MW, Nyakundi P, Gathere S, Kiptui, et al. Prevalence and determinants of heavy episodic drinking among adults in Kenya: analysis of the STEPwise survey, 2015. BMC Public Health. 2018 Nov;18(3):1–9. doi: 10.1186/s12889-018-6057-6 30400910 PMC6219062

[14] Chelogoi DN, Jonyo FO, Amadi H. The Influence of Demographic Factors in Access to Public Health Care in Kenya: A Case of Nairobi County, Kenya. Journal of Social and Political Sciences. 2020:3(2):439–454. doi: 10.31014/aior.1991.03.02.181

[15] Griffith DM, Sharma G, Holliday CS, Enyia OK, Valliere M, Semlow AR, et al. Men and COVID-19: a biopsychosocial approach to understanding sex differences in mortality and recommendations for practice and policy interventions. Preventing chronic disease. 2020 Jul 16;17:E63. doi: 10.5888/pcd17.200247 32678061 PMC7380297

[16] Bwire GM. Coronavirus: why men are more vulnerable to Covid-19 than women?. SN comprehensive clinical medicine. 2020 Jul;2(7):874–6. doi: 10.1007/s42399-020-00341-w 32838138 PMC7271824

[17] Thorp M, Balakasi KT, Mphande M, Robson I, Khan S, Stillson C, et al. Factors associated with men’s health facility attendance as clients and caregivers in Malawi: a community-representative survey. BMC Public Health. 2022 Oct 12;22(1):1904. doi: 10.1186/s12889-022-14300-8 36224573 PMC9558411

[18] Beia T, Kielmann K, Diaconu K. Changing men or changing health systems? A scoping review of interventions, services and programmes targeting men’s health in sub-Saharan Africa. International journal for equity in health. 2021 Dec;20(1):1–6 33789688 10.1186/s12939-021-01428-z PMC8011198

[19] Handcock M. “it’ll get better on its own”: Men and their resistance to seeing a doctor [Internet]. The Health Policy Partnership; 2022 [cited 2023 Jul 15]. Available from: https://www.healthpolicypartnership.com/itll-get-better-on-its-own-men-and-their-resistance-to-seeing-a-doctor/#:~:text=But%20why%20are%20many%20men

[20] WHO. Breaking Barriers: Towards More Gender-Responsive and Equitable Health Systems. 2019 Oct 18: World Health Organization. Available from: https://www.who.int/docs/default-source/documents/gender/gender-gmr-2019.pdf

[21] Parent MC, Hammer JH, Bradstreet TC, Schwartz EN, Jobe T. Men’s mental health help-seeking behaviors: An intersectional analysis. American journal of men’s health. 2018 Jan;12(1):64–73. doi: 10.1177/1557988315625776 29226771 PMC5734540

[22] Roudsari RL, Sharifi F, Goudarzi F. Barriers to the participation of men in reproductive health care: a systematic review and meta-synthesis. BMC Public Health. 2023 May 4;23(1):818. doi: 10.1186/s12889-023-15692-x 37143008 PMC10158256

[23] Haase S, Zweigenthal V, Müller A. Barriers in Access to Healthcare for Kenyan Queer Womxn and Trans Men: Findings of a Cross-Sectional Online Survey and Interviews.

[24] Dowden J, Mushamiri I, McFeely E, Apat D, Sacks J, Ben Amor Y. The impact of “male clinics” on health-seeking behaviors of adult men in rural Kenya. PloS one. 2019 Nov 21;14(11):e0224749. doi: 10.1371/journal.pone.0224749 31751377 PMC6872147

[25] Magesa DJ, Leshabari M. Perceived barriers to access available health services among men who have sex with men in Dar es Salaam, Tanzania. Tanzania Journal of Health Research. 2017 Oct 31;19(4). 10.4314/thrb.v16i2.8 26875306

[26] Hlongwa M, Jama NA, Mehlomakulu V, Pass D, Basera W, Nicol E. Barriers and facilitating factors to HIV treatment among men in a high-HIV-burdened district in KwaZulu-Natal, South Africa: a qualitative study. American Journal of Men’s Health. 2022 Sep;16(5):15579883221120987. 10.1177/15579883221120987 PMC9459474 36066024

[27] Dovel K, Dworkin SL, Cornell M, Coates TJ, Yeatman S. Gendered health institutions: examining the organization of health services and men’s use of HIV testing in Malawi. Journal of the International AIDS Society. 2020 Jun;23:e25517. doi: 10.1002/jia2.25517 32589346 PMC7319160

[28] Morrell R, Jewkes R, Lindegger G, Hamlall V. Hegemonic masculinity: reviewing the gendered analysis of men’s power in South Africa. South African Review of Sociology. 2013 Apr 1;44(1):3–21.

[29] Emslie C, Ridge D, Ziebland S, Hunt K. Men’s accounts of depression: reconstructing or resisting hegemonic masculinity?. Social science & medicine. 2006 May 1;62(9):2246–57. doi: 10.1016/j.socscimed.2005.10.017 16289741

[30] Leone JE, Rovito MJ, Mullin EM, Mohammed SD, Lee CS. Development and testing of a conceptual model regarding men’s access to health care. American journal of men’s health. 2017 Mar;11(2):262–74. doi: 10.1177/1557988316671637 27698256 PMC5675291

[31] Connell RW. Masculinities. Routledge; 2020 Jul 16.

[32] Schermerhorn NE, Vescio TK. Men’s and women’s endorsement of hegemonic masculinity and responses to COVID-19. Journal of Health Psychology. 2023 Mar;28(3):251–66. doi: 10.1177/13591053221081905 35274550 PMC9982413

[33] Shai N, Jewkes R, Nduna M, Dunkle K. Masculinities and condom use patterns among young rural South Africa men: a cross-sectional baseline survey. BMC public health. 2012 Dec;12(1):1–9. 22892159 10.1186/1471-2458-12-462 PMC3504511

[34] Hochbaum G, Rosenstock I, Kegels S. Health belief model. United states public health service. 1952;1:78–80.

[35] Turinawe EB, Rwemisisi JT, Musinguzi LK, de Groot M, Muhangi D, de Vries DH, et al. Traditional birth attendants (TBAs) as potential agents in promoting male involvement in maternity preparedness: insights from a rural community in Uganda. Reprod Health. 2016;13(1):24. doi: 10.1186/s12978-016-0147-7 26969448 PMC4788932

[36] Wambui T, Ek AC, Alehagen S. Perceptions of family planning among low-income men in Western Kenya. Int Nurs Rev. 2009;56(3):340–5. doi: 10.1111/j.1466-7657.2009.00726.x 19702808

[37] Gibore NS, Bali TA. Community perspectives: An exploration of potential barriers to men’s involvement in maternity care in a central Tanzanian community. PloS one. 2020 May 21;15(5):e0232939. doi: 10.1371/journal.pone.0232939 32437360 PMC7241761

[38] Duze MC, Mohammed IZ. Male knowledge, attitude, and family planning practices in northern Nigeria. Afr J Reprod Health. 2006;10(3):53–65. 17518131

[39] de Klerk J, Bortolani A, Meta J, Erio T, Rinke de Wit T, Moyer E. ‘It is not fashionable to suffer nowadays’: Community motivations to repeatedly participate in outreach HIV testing indicate UHC potential in Tanzania. Plos one. 2021 Dec 22;16(12):e0261408. doi: 10.1371/journal.pone.0261408 34937061 PMC8694479

[40] Marshall AI, Kantamaturapoj K, Kiewnin K, Chotchoungchatchai S, Patcharanarumol W, Tangcharoensathien V. Participatory and responsive governance in universal health coverage: an analysis of legislative provisions in Thailand. BMJ global health. 2021 Feb 1;6(2):e004117. doi: 10.1136/bmjgh-2020-004117 33602688 PMC7896578

[41] Challenges in Kenya’s Healthcare Systems–Kenya Healthcare Federation [Internet]. 2021 [cited 2023 Jul 18]. Available from: https://khf.co.ke/blog/2021/01/08/challenges-in-kenyas-healthcare-systems/

[42] Griffith DM. “Centering the margins”: moving equity to the center of men’s health research. American Journal of Men’s Health. 2018 Sep;12(5):1317–27. doi: 10.1177/1557988318773973 29749300 PMC6142151

[43] Ten arguments for why gender should be a central focus for universal health coverage advocates [Internet]. Research in Gender and Ethics (RinGs); 2014 [cited 2023 Jul 15]. Available from: https://www.ringsgenderresearch.org/wp-content/uploads/2018/07/RinGs-Ten-arguments-for-why-gender-should-be-a-central-focus-for-UHC-advocates.pdf

[44] Patton MQ. Qualitative research & evaluation methods: Integrating theory and practice. Sage publications; 2014 Oct 29.

[45] Roudsari RL, Sharifi F, Goudarzi F. Barriers to the participation of men in reproductive health care: a systematic review and meta-synthesis. BMC Public Health. 2023 May 4;23(1):818. doi: 10.1186/s12889-023-15692-x 37143008 PMC10158256

[46] Macdonald JA, Mansour KA, Wynter K, Francis LM, Rogers A, Angeles MR, et al. Men’s and boys’ barriers to health system access. A literature review. Canberra: Australian Government Department of Health. 2022.

[47] Bakibinga P, Kisia L, Atela M, Kibe PM, Kabaria C, Kisiangani I, et al. Demand and supply-side barriers and opportunities to enhance access to healthcare for urban poor populations in Kenya: a qualitative study. BMJ open. 2022 May 1;12(5):e057484. doi: 10.1136/bmjopen-2021-057484 35523490 PMC9083429

[48] Novak JR, Peak T, Gast J, Arnell M. Associations between masculine norms and health-care utilization in highly religious, heterosexual men. American journal of men’s health. 2019 Jun;13(3):1557988319856739. doi: 10.1177/1557988319856739 31184245 PMC6560804

[49] Graf N. Why workers don’t always take family or medical leave when they need to. Available from: https://www.pewresearch.org/short-reads/2017/04/04/why-workers-dont-always-take-family-or-medical-leave-when-they-need-to/

[50] Taber JM, Leyva B, Persoskie A. Why do people avoid medical care? A qualitative study using national data. Journal of general internal medicine. 2015 Mar;30:290–7. doi: 10.1007/s11606-014-3089-1 25387439 PMC4351276

[51] Camlin CS, Ssemmondo E, Chamie G, El Ayadi AM, Kwarisiima D, Sang N, et al. Men “missing” from population-based HIV testing: insights from qualitative research. AIDS care. 2016 Jun 2;28(sup3):67–73. doi: 10.1080/09540121.2016.1164806 27421053 PMC5749410

[52] Khumalo S, Mabaso M, Makusha T, Taylor M. Narratives of young black men on barriers to health care and poor health care seeking behaviours at a university setting: a qualitative study. BMC Health Services Research. 2021 Dec;21(1):1–9. 33971874 10.1186/s12913-021-06470-9 PMC8111892

[53] Mursa R, Patterson C, Halcomb E. Men’s help‐seeking and engagement with general practice: An integrative review. Journal of Advanced Nursing. 2022 Jul;78(7):1938–53. doi: 10.1111/jan.15240 35384022 PMC9322545

[54] Munisi G., Mbele S., Ngoye E., Kahwa J., Mfugale L., and Agarwal A. 2021. The Importance of Male Engagement and Male-Friendly Services for Improving Uptake of Health Services: EngenderHealth’s Experience through the USAID Boresha Afya Programs in Tanzania. Washington, DC: EngenderHealth.Available from: https://www.engenderhealth.org/wp-content/uploads/2022/02/USAID-Boresha-Afya-Tanzania-Male-Friendly-Services-brief.pdf

[55] Skovdal M, Campbell C, Madanhire C, Mupambireyi Z, Nyamukapa C, Gregson S. Masculinity as a barrier to men’s use of HIV services in Zimbabwe. Globalization and health. 2011 Dec;7(1):1–4. doi: 10.1186/1744-8603-7-13 21575149 PMC3107786

[56] Mbokazi N, Madzima R, Leon N, Lurie MN, Cornell M, Schmidt BM, et al. Health Worker Experiences of and Perspectives on Engaging Men in HIV Care: A Qualitative Study in Cape Town, South Africa. J Int Assoc Provid AIDS Care. 2020 Jan-Dec;19:2325958220935691. doi: 10.1177/2325958220935691 ; PMCID: PMC7325454 32597712 PMC7325454

[57] Robertson S, Gough B, Hanna E, Raine G, Robinson M, Seims A, et al. Successful mental health promotion with men: the evidence from ‘tacit knowledge’. Health Promotion International. 2018 Apr 1;33(2):334–44. 10.1093/heapro/daw067 27543933

[58] Cheatham CT, Barksdale DJ, Rodgers SG. Barriers to health care and health‐seeking behaviors faced by Black men. Journal of the American Academy of Nurse Practitioners. 2008 Nov;20(11):555–62. doi: 10.1111/j.1745-7599.2008.00359.x 19128339

[59] Kamran I, Tasneem Z, Parveen T, Niazi R. Family planning through the lens of men: Readiness, preferences, and challenges. Washington: Population Council, The Evidence Project; 2015. Available from: https://knowledgecommons.popcouncil.org/departments_sbsr-rh/878/

[60] Baker P, Dworkin SL, Tong S, Banks I, Shand T, Yamey G. The men’s health gap: men must be included in the global health equity agenda. Bulletin of the World Health Organization. 2014 Mar 6;92:618–20. doi: 10.2471/BLT.13.132795 25197149 PMC4147416

[61] Novak JR, Peak T, Gast J, Arnell M. Associations between masculine norms and health-care utilization in highly religious, heterosexual men. American journal of men’s health. 2019 Jun;13(3):1557988319856739. doi: 10.1177/1557988319856739 31184245 PMC6560804

[62] Chikovore J, Hart G, Kumwenda M, Chipungu GA, Desmond N, Corbett L. Control, struggle, and emergent masculinities: a qualitative study of men’s care-seeking determinants for chronic cough and tuberculosis symptoms in Blantyre, Malawi. BMC public health. 2014 Dec;14(1):1–2. doi: 10.1186/1471-2458-14-1053 25301572 PMC4200169

[63] Yousaf O, Grunfeld EA, Hunter MS. A systematic review of the factors associated with delays in medical and psychological help-seeking among men. Health psychology review. 2015 Jan 1;9(2):264–76. doi: 10.1080/17437199.2013.840954 26209212

[64] Smith J, Braunack-Mayer A, Wittert G. What do we know about men’s help-seeking and health service use?. 10.5694/j.1326-5377.2006.tb00124.x 16411874

[65] Hardee K, Croce-Galis M, Gay J. Are men well served by family planning programs?. Reproductive health. 2017 Jan 23;14(1):14. doi: 10.1186/s12978-017-0278-5 28115004 PMC5260026

[66] White A, Tod M. The need for a strategy on men’s health. Trends in Urology & Men’s Health. 2022 Mar;13(2):2–8.

