# Supplementary material for: “For a man to go to hospital, then that would be his last option”: A qualitative study exploring men’s experiences, perceptions and healthcare needs in the implementation of Universal Health Coverage in Kenya
Source: PLOS Glob Public Health. 2024 May 7;4(5):e0002925. doi: 10.1371/journal.pgph.0002925 (PMC11075886; doi:10.1371/journal.pgph.0002925)
Supplement: S1 File — (DOCX) [file pgph.0002925.s001.docx]

### S2 Appendix: FGD Guide for Men

**Facilitator’s welcome, introduction and instructions to participants:**

**Welcome** and thank you for volunteering to take part in this discussion. You have been asked to participate as your point of view is important. I realize you are busy and I appreciate your time.

**Introduction:** This focus group discussion is designed to assess health systems capacity and populations’ perceptions and needs, for Universal Health Coverage in Kenya. The study team would like to hear your views regarding access to health care, the quality of services provided and your expectations regarding the services that the community would wish to access under the UHC.

| County: | | | Moderator: | | |
| --- | --- | --- | --- | --- | --- |
| Sub-county: | | | Note taker: | | |
| Date of FGD: | | | Start Time: | | |
| Location of FGD: | | | Stop Time: | | |
| Participants at start: | | | De-brief notes | | |
| Participants at stop: | | |  |  |  |
| **Participant** | **Age** | **Sex** | **Education level** | **Religion** | **Occupation** |
| 1 |  |  |  |  |  |
| 2 |  |  |  |  |  |
| 3 |  |  |  |  |  |
| 4 |  |  |  |  |  |
| 5 |  |  |  |  |  |
| 6 |  |  |  |  |  |
| 7 |  |  |  |  |  |
| 8 |  |  |  |  |  |
| 9 |  |  |  |  |  |
| 10 |  |  |  |  |  |

1. What do you understand by the term Universal Health Coverage?
2. What are your thoughts about the government introducing the universal health coverage? How will these help the community?
3. In your opinion, what challenges do you expect to hinder the implementation of UHC?
4. In the event that the government rolls out UHC, what would be your specific needs as men who are seeking healthcare?
5. What has been your experience regarding health services provided at the facility where you seek services from?

Probe for:

*Availability of services and drugs,*

*Equipment, quality of services,*

*Staff availability and attitude,*

*Costs involved*

*Accessibility -Distance,*

1. Tell me something about the experience you have had with nurses or doctors as you seek health services.
2. How do you pay for your medical bills? (*Probe NHIF, community based insurance, OOP*)
3. Is there anything else you would like to say about men’s health issues that we have not discussed?

***We have now come to the end of our interview.***

***Thank you very much for your time.***
